# Associations between sedentary behaviour patterns and depression among people aged 60 and older in Hebei Province of China

**DOI:** 10.1186/s12889-022-12727-7

**Published:** 2022-02-11

**Authors:** Jiaqi Wang, Ruiqiang Li, Limin Zhang, Xian Gao, Meiqi Zhou, Xinjing Zhang, Yuxia Ma

**Affiliations:** 1 Department of Nutrition and Food Hygiene, School of Public Health, Hebei Medical University, Hebei Province Key Laboratory of Environment and Human Health, Shijiazhuang, China; 2 Circulating Chemical Industry Park Hospital, The First Hospital of Hebei Medical University, Shijiazhuang, China

**Keywords:** Depression, Passive sedentary behaviours, Mentally active sedentary behaviours, Older adults

## Abstract

**Background:**

Sedentary behaviours (SBs) are now considered a risk factor for depression. Older adults are sedentary most of the time and are at a high risk of depression. However, not all types of SBs have adverse effects on mental health. Passive SBs (such as watching TV) increase the risk of depression, whereas mentally active SBs (such as using the internet and reading) decrease the risk of depression. The aim of this study was to explore the associations between type of SBs (i.e., passive and mentally active SBs) and depression among people aged 60 years and older in the Hebei Province of China.

**Methods:**

This cross-sectional study used data from the baseline survey of the Community-based Cohort Study on Nervous System Diseases. A total of 2679 older adults aged ≥60 years from the Hebei Province of China were included in this study. The type and time spent on SBs were self-reported. Watching TV was defined as a passive SB, whereas internet use, reading, and social SBs (including communicating with others and playing chess) were defined as mentally active SBs. Depression was evaluated using the Geriatric Depression Scale. The maximal possible score was 30 points, and ≥ 11 points indicated depression. Logistic regression analysis was used to assess the relationship between SBs and depression. Covariates included sex, age, education, employment, smoking, alcohol consumption, sleep duration, domestic work, physical exercise, body mass index (BMI), and chronic diseases.

**Results:**

At baseline, the participants who spent two or more hours and 0 h on passive SBs (i.e., TV viewing) had a greater risk of depression (=0 h: adjusted OR = 2.09, 95% CI = 1.18–3.76; 2–3 h: OR = 2.21, 95% CI = 1.16–4.16; > 3 h: OR = 3.59, 95% CI = 1.93–6.68) than the participants who spent 1–2 h on passive SBs. The participants who spent > 1 h on mentally active SBs had a lower risk of depression (adjusted OR = 0.26, 95% CI = 0.06–0.71) than the participants who did not engage in mentally active SBs. Not all mentally active SBs were linked to depression. The participants who engaged in social SBs had a lower risk of depression (adjusted OR: 0.24, 95% CI: 0.06–0.66) than the participants who did not engage in social SBs.

**Conclusions:**

Spending 2 h or more per day on passive SBs (watching TV) was associated with a high risk of depression among people aged 60 years and older in the Hebei Province of China. Mentally active SBs (predominantly social SBs) could reduce the risk of depression. Some participants with depression probably did not watch TV. These findings suggested that spending more time on social SBs (such as communicating with others and playing chess) rather than watching TV may have important public health implications for preventing and managing depression among older Chinese adults. Moreover, society should attend to the mental health of elderly adults who do not watch TV as they may be more prone to suffer from depressive symptoms.

**Supplementary Information:**

The online version contains supplementary material available at 10.1186/s12889-022-12727-7.

## Introduction

Years lived with disability from depression disorders have increased from 9.3% in 1990 to 37.5% in 2010 worldwide due to population growth and ageing [1]. According to the World Health Organization, in 2015, more than 300 million people had depression, and this value was equivalent to 4.4% of the world’s population [2]. Older adults, especially those aged 55–74 years, have a high incidence of depression, with prevalence rates of more than 7.5% in females and 5.5% in males [2]. Depression is known to lead to health problems, such as coronary atherosclerosis, diabetes, and metabolic syndrome [3–5].

Sedentary behaviours (SBs) are defined as any waking behaviour characterized by an energy expenditure of 1.5 metabolic equivalents (METs) while in a sitting or reclining posture [6]. Evidence has shown that older men and women spend nearly 70% [7] and 65% [8], respectively, of their days on SBs. SBs are a strong risk factor for cardiovascular disease [9], type 2 diabetes [10], cancer [11, 12], and all-cause mortality [13, 14] and can cause many psychological problems, such as depression [15, 16] and anxiety [17, 18]. A small number of studies have demonstrated a positive relationship between total sedentary time and depression in older adults [19–23].

However, whether all types of SBs increase the risk of depression remains unknown. A cross-sectional study including 24,060 participants found that substituting passive SBs (such as watching TV) with mentally active SBs (such as office work) may reduce depression risk in adults [24]. A 13-year cohort study also concluded that participants who spent ≥ 3 h per day on mentally active SBs (such as sitting in a meeting and knitting/sewing) had a significantly lower risk of major depression than those who spent < 3 h per day on mentally active SBs (*p* = 0.018), [25]. In addition, other studies have reported that internet use and reading provide benefits against depression [26, 27]. One possible mechanism for this difference is that internet use represents improved social interaction [28] and minimizes loneliness [29], whereas watching TV is associated with loneliness [30]. Nevertheless, the classification of passive and mentally active SBs is not precise. Some studies have suggested that watching TV is classified as a passive SB while using computers and reading are classified as mentally active SBs [24, 31, 32].

While a few studies have examined the association of different SBs with depression in aged adults, these findings have been contradictory or mixed [32–37]. Several studies have reported that watching TV [32, 33] increases the risk of depression in senior adults. However, two other studies did not find that prolonged TV watching increased the risk of depression [34, 35]. The lack of significance of these results may be attributed to the small sample sizes (i.e., 1383 [34] and 307 [35]) in these two studies. In contrast, two other studies found that watching TV protected against depression [36, 37]. However, a Nepalese study looked at TV watching TV and radio listening as a whole and could not determine which activity reduced the risk of depression. In addition, unlike in other countries, Nepal has more religious TV programs, which are popular with people [38]. Religion may be good for mental health in Nepal, but prolonged TV watching does not necessarily decrease the risk of depression. Another study included only 252 participants in a community of Taichung City [37]. This study found that watching TV on weekends reduced the risk of depression. However, the sample size was too small to be representative.

Only three studies examined the relationship between mentally active SBs and depression in older adults, but these studies had some problems [32, 33, 37]. One study found that older adults using the internet had lower depressive symptoms than those who did not engage in this activity, but reading the newspaper was not associated with depressive symptoms [33]. Andrade-Gomez et al. looked at reading, using the computer, and commuting and studied their relationship with depression as a whole rather than exploring their separate effects [32]. The third study, which examined the relationships between three mentally positive activities (reading newspapers and magazines, listening to radio, and engaging with internet) and depression, included only 252 participants [37].

In China, a meta-analysis showed that the pooled prevalence of depressive symptoms in elderly adults aged ≥ 60 years was 23.6% (95% CI = 20.3–27.2%) and has increased steadily from 1987 to 2012 [39]. The total sedentary time of older Chinese adults increased by 0.58 h per day from 2002 to 2012 [40]. It is essential to clarify the relationship between SBs and depression in older Chinese adults. However, studies on the relationship between SBs and depression in elderly Chinese individuals are unavailable. Moreover, how different SBs affect depression in older adults remains unclear. Accordingly, the aim of the present study was to explore the associations between SBs (passive and mentally active SBs) and depression in people aged 60 years and older in the Hebei Province of China. Second, to explore whether different mentally positive SBs increased the risk of depression, we analysed the relationships between depression and using the internet, reading, and other SBs.

## Methods

### Study design and participants

The study used data from the Community-based Cohort Study on Nervous System Diseases (No. 2017YFC0907701) [41–43], a population-based longitudinal survey designed to establish community population cohorts of individuals with epilepsy, Alzheimer’s disease (AD), and Parkinson’s disease (PD); to explore disease risk factors; and to provide the scientific basis for realizing effective three-level prevention strategies at the community and individual levels. This study included participants aged > 1 year without epilepsy or aged ≥ 55 years without AD and PD. The survey selected sites in Hebei, Zhejiang, Shanxi, and Hunan provinces. Two cities and two counties in each province were randomly selected. Urban and suburban neighbourhoods within cities and townships and villages within counties were randomly chosen. Volunteers and their families took part in the survey at each site, and those who did not meet the requirements based on the inclusion criteria were excluded.

This cross-sectional study used data from the baseline survey established in 2018. The participants in this study were selected only from Hebei Province only. A total of two urban areas, two suburban areas, two townships, and one village were randomly selected from two cities (i.e., Shijiazhuang and Tangshan) and two counties (i.e., Cangzhou and Xingtai). A total of 6720 people participated in the survey, of which 3254 people were aged 60 years and above. Participants with incomplete data regarding SBs (*n* = 547) and vague answers to the depression questionnaire (*n* = 28) were excluded from the analyses. The remaining 2679 participants were included in the current study. Compared with the included participants, the excluded participants had higher depressive symptoms and lower domestic work, physical exercise, and body mass index (BMI) levels (Supplementary Table 1). All personnel involved in data collection were adequately trained before the commencement of fieldwork.

### SBs

SBs were measured using a questionnaire from the China Health and Nutrition Survey. The survey questionnaire was designed by an interdisciplinary group of social and biomedical scientists with extensive experience in survey research and construction related to their respective fields. The SBs in this questionnaire have been reported in other studies [44, 45]. The participants reported the average hours each day during the week and on weekends that they were sitting and engaged in in four activities: watching TV, using the internet, reading (books, newspapers, and magazines), and social SBs (e.g., communicating with others and playing chess). Based on previous findings [24, 31, 32], watching TV was classified as a passive SB, whereas the others were classified as mentally active SBs. The average daily hours spent on a particular sedentary activity was calculated as ([time spent on a particular sedentary activity time on the weekday × 5] + [time spent on a particular sedentary activity on the weekend × 2])/7. With reference to previous findings [34, 46, 47], the time spent on passive SBs was categorized into five groups (0, 0 to ≤ 1, 1 to 2, 2 to 3, and > 3 h per day), and the time spent on mentally active SBs was categorized into three groups (0, 0 to ≤1, and > 1 h per day).

These different kinds of mentally active SBs might have distinct associations with depression. The Internet use and social SBs were categorized into three groups (0, 0 to ≤1, and > 1 h per day). Reading was categorized into three groups (0, 0 to ≤ 0.5, and > 0.5 h per day).

### Depression

Depression was assessed using the Geriatric Depression Scale (GDS-30), which consisted of 30 items with 0–1 points for each item [48]. The GDS-30 has been used in previous studies [49–51]. The total score could range from 0 to 30 points, and the cut-off value was 11. A score < 11 indicated no depressive symptoms and a score ≥ 11 indicated depressive symptoms. Most previous studies used 11 as the cut-off value [41, 52, 53]. A GDS-30 validity survey in a large Chinese ageing population found that with 11 as the cut-off value, the Kuder–Richardson coefficients for a cognitively normal elderly group, a mild cognitive impairment screening group, and the whole population were 0.834, 0.821, and 0.840, respectively [41]. Other studies have also demonstrated the instrument’s validity [54–56].

### Covariates

The analyses were adjusted for covariates including sex, age, education, employment (yes/ no), smoking, alcohol consumption in the previous year (2017), sleep duration, domestic work, physical exercise, BMI, and chronic diseases [22, 32–34]. Height was measured using a stable stadiometer (Seca, Germany) with 0.1 cm precision, and the participants were required to remove their shoes. Weight was measured using an electronic scale with 0.5 kg precision, and participants were asked to remove their shoes and minimize clothing. BMI was calculated by dividing body weight in kg by height squared in m^2^. BMI was divided into two groups (< 24 and ≥ 24 kg/m^2^) following the Guidelines for the Prevention and Control of Overweight and Obesity in Chinese Adults (2003).

Physical exercise and domestic work were measured using the China Health and Nutrition Survey questionnaire that has been reported in other studies [57, 58]. The participants reported the average hours each day during the week and on weekends that they engaged in a particular sport (including martial arts; running or swimming; gymnastics, dancing, or acrobatics; walking, football, basketball, or tennis; table tennis or tai chi). Investigators also asked the participants how much domestic work (including buying food, cooking, doing laundry, washing or ironing clothes by using hands, and cleaning rooms) they did each day. According to the Compendium of Physical Activities [59] and a previous study [60], physical exercise and domestic work were assessed against general or moderate criteria, and METs/hour were estimated as follows: 10.0 for martial arts; 7.0 for running or swimming; 4.5 for gymnastics, dancing, or acrobatics; 3.5 for walking; 7.0 for playing football, basketball, or tennis; 3.75 for playing badminton or volleyball; 4.0 for playing table tennis or tai chi; 2.3 for buying food; 2.25 for cooking; 2.0 for doing laundry; 2.3 for washing or ironing clothes with hands; and 3.0 for cleaning rooms. The MET-hours/day for each particular sport or type of domestic work was calculated as (specific sport/domestic work time × hours spent per day).

Measures of other covariates, including sex, age (≤ 65, 65–70,70–75, > 75 years), education (primary and below or secondary and above), employment (yes or no), smoking (current/previous smoking or nonsmoking), alcohol consumption (yes or no) in the previous year (2017), sleep duration (≤ 6, 6 to 9, or ≥ 9 h), and chronic diseases (0, 1, or ≥ 2), were obtained by conducting a face-to-face interview. Chronic diseases included hypertension, diabetes, myocardial infarction, and stroke; each was assigned a score of 1 for a total score range of 0–4 points. Higher scores indicated more chronic diseases [7]. The history of the disease was determined by asking the participants whether their doctor had given them a particular disease diagnosis.

### Statistical analysis

Continuous variables are presented as the mean ± standard deviation, and categorical variables are expressed as percentages (%). Chi-squared tests for categorical variables and t tests or Wilcoxon rank tests for continuous variables were performed to compare the depression and nondepression groups. Multivariable logistic regression analyses were used to estimate odds ratios (ORs) with 95% confidence intervals (CIs) for the association between depression and SBs (passive and mentally active SBs). In all models, the reference group for passive SBs was 1–2 h, and that for mentally active SBs was 0 h. Three models were built. Model 1 adjusted for sex, age, education, work, smoking, alcohol consumption, BMI, and chronic diseases. Next, Model 2 adjusted Model 1 covariates and sleep duration, domestic work, and physical exercise. Finally, Model 3 adjusted Model 2 covariates and the other category of SBs (for example, adjusted for mentally active SBs when exploring the relationship between passive SBs and depression).

To explore different mentally positive SBs that might increase the risk of depression, we analysed the relationship between depression and using the internet, reading, and other SBs. Multivariable logistic regression analyses were used, and the reference group for internet use, reading, and other SBs was 0 h in all models. Model 1 and Model 2 were adjusted as described above. Model 3 adjusted Model 2 covariates and each other type of SBs (for example, adjusted for passive SBs, reading, and social SBs when exploring the relationship between using the internet and depression).

Considering that missing values among the covariate measures might influence the results, a sensitivity analysis was conducted to investigate the association between SB types and depression after removing variables containing missing values.

Statistical significance was set at two-sided *p* <  0.05. All analyses were performed using the R software (version 4.0.0).

## Results

Sample characteristics are presented in Table 1. Of the 2679 participants, 139 (5.19%) had depression. Compared with the participants who scored high on the GDS-30 scale, the participants who scored low were older, smoked less, consumed less energy with domestic work and physical exercise, spent more time on passive SBs, and had lower BMI.

**Table 1:** Characteristics of the study sample

	Non-depression	Depression	***p*** value ^**a**^
	*n* = 2540	*n* = 139	
**Age (years)**			
≤ 65	763 (30.0)	34 (24.5)	0.030^*^
65–70	639 (25.2)	25 (18.0)	
70–75	646 (25.4)	44 (31.7)	
> 75	492 (19.4)	36 (25.8)	
**Sex**			
Men	1096 (43.1)	50 (36.0)	0.115
Women	1444 (56.9)	89 (64.0)	
**Smoke**			
Current/Previous	127 (16.2)	11 (8.0)	0.014^*^
Never	2125 (83.8)	411 (92.0)	
**Drink (2017)**			
No	2135 (84.3)	123 (89.1)	0.158
Yes	398 (15.7)	15 (10.9)	
**Education**			
Primary and below	1628 (64.3)	92 (68.1)	0.416
Secondary and above	903 (35.7)	43 (31.9)	
**Employment**			
Yes	131 (8.7)	8 (5.8)	0.299
No	2320 (91.3)	220 (94.2)	
**Chronic diseases** ^**b**^			
0	1466 (58.0)	74 (55.7)	
1	860 (34.0)	45 (33.8)	0.570
2	202 (8.0)	14 (10.5)	
**Domestic work (MET-h/d)**	3.9 ± 3.7	3.1 ± 3.5	0.021^*^
**Physical exercise (MET-h/d**)	2.2 ± 3.7	1.7 ± 3.3	0.017^*^
**Passive SBs (h/d)**			
= 0	450 (17.7)	37 (26.6)	< 0.001^*^
> 0–≤ 1	654 (25.7)	23 (16.5)	
1–2	842 (33.1)	27 (19.4)	
2–3	330 (13.0)	26 (18.7)	
> 3	264 (10.4)	26 (18.7)	
**Mentally active SBs (h/d)**			
= 0	1944 (76.5)	116 (83.5)	0.010^*^
> 0–≤1	236 (9.3)	15 (10.8)	
> 1	360 (14.2)	8 (5.7)	
**Sleep duration (h/d)**			
≤ 6	744 (29.3)	52 (37.4)	0.088
6–9	1594 (62.8)	80 (57.6)	
≥ 9	200 (7.9)	7 (5.0)	
**BMI (kg/m**^**2**^**)**			
< 24	929 (39.9)	68 (51.9)	0.008^*^
≥ 24	1400 (60.1)	63 (48.1)	

*BMI, body mass index*

Covariates were missing for BMI (*n* = 200), education (*n* = 13), smoke (*n* = 5), drink (*n* = 8), chronic diseases (*n* = 16), domestic work (*n* = 144), physical exercise (*n* = 14), sleep (*n* = 2)

^a^Values are means ± SDs or n (%). The difference in sample characteristics was tested by Chi-squared tests and t-tests or Wilcoxon rank test for categorical and continuous variables respectively

^b^Chronic diseases included hypertension, diabetes, myocardial infarction, and stroke, each of which was assigned a score of 1

^*^p <  0.05

Table 2 displays the association of passive SBs and mentally active SBs with depression. After the final model adjustment, depression was associated with 3.59 (95% CI = 1.93–6.68) times higher odds among participants who spent 3 or more hours on passive SBs and 2.21 (95% CI = 1.16–4.16) times higher odds among participants who spent 2–3 h in comparison with those who spent 1–2 h on passive SBs. The participants who spent 0 h on passive SBs had an increased risk of depression compared to those who spent 1–2 h (adjusted OR = 2.09, 95% CI = 1.18–3.76). The participants who spent > 1 h on mentally active SBs had a decreased risk of depression (adjusted OR = 0.24, 95% CI = 0.06–0.67) compared with those without mentally active SBs after the final model adjustment.

**Table 2:** Associations between depression and sedentary behaviors (SBs) assessed by multivariable logistic

	Model 1		Model 2		Model 3	
	OR (95% CI)	***p*** value	OR (95% CI)	***p*** value	OR (95% CI)	***p*** value
**Passive SBs (h/d)**						
= 0	2.49 (1.46,4.33)	0.001^*^	1.99 (1.12,3.57)	0.019	2.09 (1.18,3.76)	0.012^*^
> 0–≤ 1	0.96 (0.51,1.78)	0.899	0.98 (0.52,1.84)	0.955	0.93 (0.49,1.75)	0.829
1–2	Ref		Ref		Ref	
2–3	2.55 (1.39,4.64)	0.002^*^	2.21 (1.16,4.15)	0.014	2.21 (1.16,4.16)	0.014^*^
> 3	3.62 (2.01,6.53)	< 0.001^*^	3.51 (1.89,6.51)	< 0.001^*^	3.59 (1.93,6.68)	< 0.001^*^
**Mentally active SBs (h/d)**						
= 0	Ref		Ref		Ref	
> 0–≤ 1	1.05 (0.54,1.87)	0.880	1.22 (0.61,2.28)	0.550	1.54 (0.74,2.94)	0.219
> 1	0.38 (0.16,0.78)	0.016^*^	0.46 (0.19,0.96)	0.059	0.26 (0.06,0.71)	0.024^*^

Model 1 adjusted for sex, age, education, employment, smoking, alcohol consumption in the past year, body mass index, chronic diseases

Model 2 adjusted Model 1 covariates and sleep duration, domestic work and physical exercise

Model 3 adjusted Model 2 covariates and the other category of SBs (for example, adjusted for mentally active SBs when exploring the relationship between passive SBs and depression)

*p < 0.05

Compared with the participants who did not participate in social SBs, the participants who spent > 1 h had a decreased risk of depression (adjusted for OR = 0.24, 95% CI = 0.06–0.66) after the final model adjustment. Spending more than 0.5 h on reading (adjusted OR = 1.09, 95% CI = 0.32–2.86) and more than one hour on using the internet (adjusted OR = 1.12, 95% CI = 0.31–3.08) had no association with depression compared with participants who did not take part in these activities after the final model adjustment (Table 3).

**Table 3:** Associations between depression and different sedentary mentally active behaviors (SBs) assessed by multivariable logistic

	Model 1		Model 2		Model 3	
	OR (95% CI)	***p*** value	OR (95% CI)	***p*** value	OR (95% CI)	***p*** value
**Using the internet (h/d)**						
= 0	Ref		Ref		Ref	
> 0–≤ 1	0.24 (0.01,1.13)	0.164	0.34 (0.02,1.60)	0.287	0.30 (0.02,1.48)	0.248
> 1	1.17 (0.34,3.04)	0.777	1.38 (0.40,3.70)	0.560	1.12 (0.31,3.08)	0.842
**Reading (h/d)**						
= 0	Ref		Ref		Ref	
> 0–≤ 0.5	0.60 (0.10,2.03)	0.494	0.78 (0.12,2.69)	0.743	0.65 (0.10,2.34)	0.576
> 0.5	0.84 (0.25,2.12)	0.743	1.09 (0.32,2.81)	0.870	1.09 (0.32,2.86)	0.874
**Social SBs (h/d)**						
= 0	Ref		Ref		Ref	
> 0–≤ 1	1.44 (0.59,3.03)	0.371	1.42 (0.53,3.18)	0.439	1.56 (0.57,3.59)	0.340
> 1	0.23 (0.06,0.62)	0.013^*^	0.27 (0.06,0.74)	0.028	0.24 (0.06,0.66)	0.017^*^

Model 1 adjusted for sex, age, education, employment, smoking, alcohol consumption in the past year, body mass index, chronic diseases

Model 2 adjusted Model 1 covariates and sleep duration, domestic work and physical exercise

Model 3 adjusted Model 2 covariates and each other type of SBs (for example, adjusted for passive SBs, reading books and newspaper, and other SBs when exploring the relationship between using the Internet and depression)

*p < 0.05

The sensitivity analysis results are presented in supplemental tables (Supplementary Tables 2–4). The participants with incomplete covariate data were excluded, and 2291 participants were eventually included. The logistic regression analysis with these participants found that the results did not change.

## Discussion

This study found a positive correlation between prolonged TV viewing (a passive SB) and depression, and this finding was consistent with those in previous studies [21, 33, 47]. Hamer et al. performed a cross-sectional study and found that TV viewing time (≥ 6 h vs. < 2 h) was associated with high depressive symptoms (β = 0.49, 95% CI = 0.63–0.35) [33]. Another cross-sectional study was conducted among 42,469 people in low- and middle-income countries, including 14,585 older adults over 65 years old [21]. In this older group, those who watched TV for 8 or more hours had a 1.72-fold increased risk of depression than those who watched TV for less than 8 h a day. A study also found that women who spent 21 or more hours/week watching TV had a 1.13-fold increased risk of depression than those who spent 0–1 h/week among 49,821 older American women from the Nurses’ Health study who were followed over 10 years [47]. Several mechanisms could explain the relationship between TV watching and depression. First, older persons experience reduced social support, increased social isolation, and increased loneliness due to changes in living circumstances [61]. Watching TV, the main way to compensate for the loss of social connections, increases loneliness [30]. Loneliness can lead to socially ineffective, passive, and negative attitudes towards the self and others and a sense of hopelessness, and loneliness has been closely associated with depression [62]. Second, a systematic review showed that prolonged engagement in SBs increases levels of inflammatory cytokines [63]. Inflammation is one of the main pathways leading to depression [64].

The present study indicated that spending no time on passive SBs was associated with increased depression. A cross-sectional survey based on 60,202 adults from Brazil’s National Health Survey showed similar results and found that less than 1 h of TV viewing was associated with increased depression [46]. However, this association was not found among older adults in the stratified analysis by age. A Brazilian study found that people who watched less TV were more likely to be without a degree. Not having a degree has been associated with low socioeconomic status, which has been associated with depression. Although the present study found a higher prevalence of having primary school and below among the participants with depression risk and low TV viewing, a similar result was also found among the participants with depression risk and high TV viewing (0: 78.4%, 0 to ≤1 h: 38.1%, 1 to 2 h: 56.0%, 2 to 3 h: 80.8%, and ≥ 3 h: 76.9%; *p* = 0.031). Therefore, the lack of TV viewing might not have been a measure-of-proxy for a low socioeconomic status in this study. The high risk of depression among the participants who did not watch TV might have been linked to the apathy associated with depression. As a prominent symptom of depression in older adults [65], apathy manifests as lack of interest and emotional flatness [66], causing the elderly individuals to lose interest in watching TV. One study also showed that participants with major depression watched less TV than participants without a diagnosis of major depression [34]. The present study also found that 26.7% of participants with depressive symptoms did not watch TV at all.

Our results suggested that engaging in mentally active SBs (predominantly social SBs) could decrease the risk of depression. A previous study found a negative correlation between social SBs and psychological stress [67]. Social SBs, such as interacting with others and playing chess, are good ways to build social connections and prevent depression. However, reading or using the internet had no association with depression in this study. A cross-sectional study found that participants using the internet reported low depressive symptoms (β = − 0.58, 95% CI = − 0.50 to − 0.66), but their longitudinal study did not find such an association [33]. Another study found only that SBs (such as reading, using the computer, and commuting) have a protective effect on depression in women and not in men [32]. Few speculations about the link between these two factors have been were put forward. First, some studies have suggested that using the internet and reading can improve cognitive function [26, 27] and that TV viewing can increase the risk of cognitive dysfunction [26]. Therefore, one study speculated that certain mentally positive SBs might counterbalance the adverse effects of harmful sitting on the brain and mental health [32]. However, whether this prediction is correct and the specific underlying mechanisms need further research. Second, another study found that using the internet to compensate for social isolation was associated with a reduced risk of depression in older adults [28]. A study conducted with older Chinese adults also found that internet use can improve life satisfaction [68]. Therefore, increased social interactions in older adults (including internet use, communicating with others, and playing chess) appear to reduce the risk of depression.

### Strengths and limitations

The strengths of this study are as follows. First, this study explored the associations between different types of SBs and depression. Second, few studies have adjusted for sleep duration and domestic work as confounders. As part of daily life, sleep duration and domestic work affected the relationship between SBs and depression.

This study had some limitations. First, this study was a cross-sectional study and could not determine the direction of the relationships between SBs and depression. Second, the study used self-reported SBs, which could introduced bias. Future research using tools, such as accelerometers, which can objectively measure sedentary time, is recommended. Third, the GDS-30, a self-assessed depression screening tool, does not represent a clinical diagnosis and may result in a certain number of false-positive and false-negative results. An international diagnostic interview or semiquantitative scale is suggested to be used to diagnose depression. Fourth, although we adjusted for multiple confounding variables, the effects of residual or other unknown confounding factors cannot be excluded. Finally, although this study explored the associations between time spent on passive SBs and depression, this association cannot be generalized to other types of SBs.

## Conclusions

This cross-sectional study showed that spending two or more hours/day on passive SBs (e.g., watching TV) seems to be associated with a high risk for depression among people aged 60 years and older in Hebei Province, China. Mentally active SBs (predominantly social SBs) could reduce the risk of depression. Some participants with depression probably did not watch TV. These findings suggest that spending more time on social SBs (such as communicating with others and playing chess) rather than watching TV may have important public health implications for preventing and managing depression among older Chinese adults. Moreover, society should attend to the mental health of elderly adults who do not watch TV, and they may be more prone to suffer from depressive symptoms.

## Supplementary Information

**Additional file 1: Supplementary Table 1.** The characteristics of the participants excluded. **Supplementary Table 2.** Characteristics of the study sample. **Supplementary Table 3.** Associations between depression and sedentary behaviors (SBs) assessed by multivariable logistic. **Supplementary Table 4.** Associations between depression and different mentally active sedentary behaviors (SBs) assessed by multivariable logistic.

## Data Availability

The data that support the findings of this study are available from the corresponding author upon reasonable request.

## Abbreviations

SBs: Sedentary behaviors
METs: Metabolic equivalents
GDS-30: Geriatric Depression Scale
AD: Alzheimer’s disease
PD: Parkinson’s disease
ORs: Odds ratios
CIs: Confidence intervals
BMI: Body mass index
