# Supplementary material for: Associations between sedentary behaviour patterns and depression among people aged 60 and older in Hebei Province of China
Source: BMC Public Health. 2022 Feb 11;22:283. doi: 10.1186/s12889-022-12727-7 (PMC8840782; doi:10.1186/s12889-022-12727-7)
Supplement: Supplementary file 1 — Additional file 1: Supplementary Table 1. The characteristics of the participants excluded. Supplementary Table 2. Characteristics of the study sample. Supplementary Table 3. Associations between depression and sedentary behaviors (SBs) assessed by multivariable logistic. Supplementary Table 4. Associations between depression and different mentally active sedentary behaviors (SBs) assessed by multivariable logistic. [file 12889_2022_12727_MOESM1_ESM.docx]

| **Supplementary table 1** The characteristics of the participants excluded | | | | | | |  |
| --- | --- | --- | --- | --- | --- | --- | --- |
|  | **Included participants** | **Excluded participants** | | | | ***p* value ^a^** |  |
|  | **n=2679** | **n=575** | | | |  |  |
| **Depression** | 139 (5.2) | 32 (8.5) | | | | 0.013 |  |
| **Age (years)** |  |  | | | |  |  |
| ≤65 | 797 (29.7) | 168 (29.2) | | | | 0.296 |  |
| 65-70 | 664 (24.8) | 156 (27.1) | | | |  |  |
| 70-75 | 690 (25.8) | 129 (22.4) | | | |  |  |
| >75 | 528 (19.7) | 122 (21.3) | | | |  |  |
| **Sex** |  |  | | | |  |  |
| Men | 1146 (42.8) | 270 (47.0) | | | | 0.074 |  |
| Women | 1533 (57.2) | 305 (53.0) | | | |  |  |
| **Smoke** |  |  | | | |  |  |
| Current/previous | 422 (15.8) | 59 (14.4) | | | | 0.503 |  |
| Never | 2252 (84.2) | 352 (85.6) | | | |  |  |
| **Drink (2017)** |  |  | | | |  |  |
| No | 2258 (84.5) | 364 (89.0) | | | | 0.022 |  |
| Yes | 413 (15.5) | 45 (11.0) | | | |  |  |
| **Education** |  |  | | | |  |  |
| Primary and below | 1720 (64.5) | 269 (66.3) | | | | 0.530 |  |
| Secondary and above | 946 (35.5) | 137 (33.7) | | | |  |  |
| **Employment** |  | |  |  |  |  |  |
| Yes | 228 (8.5) | 36 (8.7) | | | | 0.946 |  |
| No | 2451 (91.5) | 377 (91.3) | | | |  |  |
| **Chronic diseases^b^** |  |  | | | |  |  |
| 0 | 1540 (57.9) | 260 (65.3) | | | |  |  |
| 1 | 905 (34.0) | 112 (30.7) | | | | 0.002 |  |
| 2 | 216 (8.1) | 16 (4.0) | | | |  |  |
| **Domestic work (MET. h/d)** | 3.9±3.7 | 2.7±3.3 | | | | <0.001^*^ |  |
| **Physical exercise (MET. h/d)** | 2.2±3.7 | 0.4±1.8 | | | | <0.001^*^ |  |
| **Sleep duration(h/d)** |  |  | | | |  |  |
| ≤6 | 796 (29.7) | 106 (27.8) | | | | 0.724 |  |
| 6-9 | 1674 (62.5) | 245 (64.1) | | | |  |  |
| ≥9 | 207 (7.3) | 31 (8.1) | | | |  |  |
| **BMI (kg/m^2^)** |  |  | | | |  |  |
| <24 | 997 (40.5) | 202 (47.1) | | | | 0.013^*^ |  |
| ≥24 | 1463 (59.5) | 227 (52.9) | | | |  |  |
| *BMI, body mass index.*  ^a^ Values are means ± SDs or n (%). The difference in sample characteristics was tested by Chi-squared tests and t-tests or Wilcoxon rank test for categorical and continuous variables respectively.  ^b^ Chronic diseases included hypertension, diabetes, myocardial infarction, and stroke, each of which was assigned a score of 1.  ^*^*p* ≤0. 05. | | | | | | |  |

| **Supplementary table 2** Characteristics of the study sample | | | | | | |  |
| --- | --- | --- | --- | --- | --- | --- | --- |
|  | **Non-depression** | | **Depression** | | | ***p* value ^a^** |  |
|  | **n=2179** | | **n=112** | | |  |  |
| **Age (years)** |  | |  | | |  |  |
| ≤ 65 | 671 (30.8) | | 28 (25.0) | | | 0.020^*^ |  |
| 65–70 | 564 (25.9) | | 19 (17.0) | | |  |  |
| 70–75 | 539 (24.7) | | 36 (32.1) | | |  |  |
| > 75 | 405 (18.6) | | 29 (25.9) | | |  |  |
| **Sex** |  | |  | | |  |  |
| Men | 958 (44.0) | | 41 (36.6) | | | 0.152 |  |
| Women | 1221 (56.0) | | 71 (63.4) | | |  |  |
| **Smoke** |  | |  | | |  |  |
| Current/previous | 348 (16.0) | | 11 (9.8) | | | 0.107 |  |
| Never | 1831 (84.0) | | 101 (90.2) | | |  |  |
| **Drink (2017)** |  | |  | | |  |  |
| No | 1832(84.1) | | 98 (87.5) | | | 0.403 |  |
| Yes | 347(15.9) | | 14 (12.5) | | |  |  |
| **Education** |  | |  | | |  |  |
| Primary and below | 1389 (63.7) | | 73 (65.2) | | | 0.836 |  |
| Secondary and above | 790 (36.3) | | 39 (34.8) | | |  |  |
| **Employment** |  |  | |  |  |  |  |
| Yes | 200 (9.2) | | 7 (6.2) | | | 0.376 |  |
| No | 1979 (90.8) | | 105 (93.8) | | |  |  |
| **Chronic diseases ^b^** |  | |  | | |  |  |
| 0 | 1279 (58.7) | | 64 (57.1) | | |  |  |
| 1 | 736 (33.8) | | 38 (33.9) | | | 0.852 |  |
| 2 | 164 (7.5) | | 10 (9.0) | | |  |  |
| **Domestic work (MET. h/d)** | 3.8±3.7 | | 3.0±3.0 | | | 0.056 |  |
| **Physical exercise (MET. h/d)** | 2.2±3.7 | | 1.9±3.6 | | | 0.161 |  |
| **Passive SBs (h/d)** |  |  | |  |  |  |  |
| =0 | 377 (17.3) | | 30 (26.8) | | | < 0.001^*^ |  |
| > 0–≤ 1 | 577 (26.5) | | 18 (16.1) | | |  |  |
| 1–2 | 723 (33.2) | | 23 (20.5) | | |  |  |
| 2–3 | 278 (12.8) | | 19 (17.0) | | |  |  |
| > 3 | 224 (10.2) | | 22 (19.6) | | |  |  |
| **Mentally active SBs (h/d)** |  |  | |  |  |  |  |
| =0 | 1682 (77.2) | | 93 (83.0) | | | 0.047^*^ |  |
| > 0–≤ 1 | 195 (8.9) | | 12 (10.7) | | |  |  |
| > 1 | 302 (13.9) | | 7 (6.3) | | |  |  |
| **Sleep duration (h/d)** |  | |  | | |  |  |
| ≤ 6 | 643 (29.5) | | 44 (39.3) | | | 0.064 |  |
| 6–9 | 1373 (63.0) | | 63 (56.3) | | |  |  |
| ≥ 9 | 163 (7.5) | | 5 (4.6) | | |  |  |
| **BMI (kg/m^2^)** |  | |  | | |  |  |
| < 24 | 878 (40.3) | | 57 (50.9) | | | 0.033^*^ |  |
| ≥ 24 | 1301 (59.7) | | 55 (49.1) | | |  |  |
| *BMI, body mass index.*  ^a^ Values are means ± SDs or n (%). The difference in sample characteristics was tested by Chi-squared tests and t-tests or Wilcoxon rank test for categorical and continuous variables respectively.  ^b^ Chronic diseases included hypertension, diabetes, myocardial infarction, and stroke, each of which was assigned a score of 1.  ^*^*p* < 0. 05. | | | | | | |  |

| **Supplementary table 3** Associations between depression and sedentary behaviors (SBs) assessed by multivariable logistic | | | | | | |
| --- | --- | --- | --- | --- | --- | --- |
|  | **Model 1** | | **Model 2** | | **Model 3** | |
|  | **OR (95% CI)** | ***p* value** | **OR (95% CI)** | ***p* value** | **OR (95% CI)** | ***p* value** |
| **Passive SBs (h/d)** |  |  |  |  |  |  |
| = 0 | 2.31 (1.31,4.11) | 0.004^*^ | 1.99 (1.12,3.57) | 0.019 | 2.09 (1.18,3.76) | 0.012^*^ |
| > 0–≤ 1 | 0.99 (0.52,1.85) | 0.967 | 0.98 (0.52,1.84) | 0.955 | 0.93 (0.49,1.75) | 0.829 |
| 1–2 | Ref | | Ref | | Ref | |
| 2–3 | 2.25 (1.19,4.22) | 0.011 | 2.21 (1.16,4.15) | 0.014 | 2.21 (1.16,4.16) | 0.014 |
| > 3 | 3.24 (1.76,5.98) | < 0.001^*^ | 3.51 (1.89,6.51) | <0.001^*^ | 3.59 (1.93,6.68) | <0.001^*^ |
| **Mentally active SBs (h/d)** |  |  |  |  |  |  |
| = 0 | Ref | | Ref | | Ref | |
| > 0–≤ 1 | 1.06 (0.54,1.94) | 0.852 | 1.22 (0.61,2.28) | 0.550 | 1.54 (0.74,2.94) | 0.219 |
| > 1 | 0.40 (0.17,0.83) | 0.024^*^ | 0.46 (0.19,0.96) | 0.059 | 0.26 (0.06,0.71) | 0.024^*^ |
| Model 1 adjusted for sex, age, education, employment, smoking, alcohol consumption in the past year, body mass index, chronic diseases.  Model 2 adjusted Model 1 covariates and sleep duration, domestic work and physical exercise.  Model 3 adjusted Model 2 covariates and the other category of SBs sedentary behaviors for example, adjusted for mentally active SBs when exploring the relationship between passive SBs and depression).  **p* < 0.05. | | | | | | |

| **Supplementary table 4** Associations between depression and different mentally active sedentary behaviors (SBs) assessed by multivariable logistic | | | | | | | |
| --- | --- | --- | --- | --- | --- | --- | --- |
|  | **Model 1** | | **Model 2** | | **Model 3** | | |
|  | **OR (95% CI)** | ***p* value** | **OR (95% CI)** | ***p* value** | **OR (95% CI)** | ***p* value** | |
| **Using the internet (h/d)** |  |  |  |  |  |  | |
| = 0 | Ref | | Ref | | Ref | | |
| > 0–≤ 1 | 0.27 (0.02,1.26) | 0.197 | 0.34 (0.02,1.60) | 0.287 | 0.30 (0.02,1.48) | 0.248 | |
| > 1 | 1.25 (0.36,3.28) | 0.680 | 1.38 (0.40,3.70) | 0.560 | 1.12 (0.31,3.08) | 0.842 | |
| **Reading (h/d)** |  |  |  |  |  |  | |
| = 0 | Ref | | Ref | | Ref | | |
| > 0–≤ 0.5 | 0.65 (0.10,2.17) | 0.553 | 0.78 (0.12,2.69) | 0.743 | 0.65 (0.10,2.34) | 0.576 | |
| > 0.5 | 0.90 (0.27,2.28) | 0.844 | 1.09 (0.32,2.81) | 0.870 | 1.09 (0.32,2.86) | 0.874 | |
| **Social SBs (h/d)** |  |  |  |  |  |  | |
| = 0 | Ref | | Ref | | Ref | | |
| > 0–≤ 1 | 1.37 (0.52,3.01) | 0.479 | 1.42 (0.53,3.18) | 0.439 | 1.56 (0.57,3.59) | | 0.340 |
| > 1 | 0.24 (0.06,0.65) | 0.016^*^ | 0.27 (0.06,0.74) | 0.028^*^ | 0.24 (0.06,0.66) | | 0.017^*^ |
| Model 1 adjusted for sex, age, education, employment, smoking, alcohol consumption in the past year, body mass index, chronic diseases.  Model 2 adjusted Model 1 covariates and sleep duration, domestic work and physical exercise.  Model 3 adjusted Model 2 covariates and each other type of SBs (for example, adjusted for passive SBs, reading books and newspapers, and other SBs when exploring the relationship between using the internet and depression).  **p* < 0. 05. | | | | | | | |
